# Contrast-associated acute kidney injury (CA-AKI) according to the type and dose of contrast in contrast-enhanced radiographic examinations. preliminay results of the nefrocon study

**DOI:** 10.1186/2197-425X-3-S1-A64

**Published:** 2015-10-01

**Authors:** C Gomez-Gonzalez, S Mas-Font, MD Herrera-Rojas, F Sanchez Moran, E Gomez, C Mudarra, V Enciso

**Affiliations:** Hospital Infanta Luisa, Sevilla, Spain; Hospital General de Castellon, Castellon, Spain; Hospital Universitario Valme, Sevilla, Spain; Hospital La Plana (Vila-Real), Castellón, Spain; Fundación Jiménez Díaz, Madrid, Spain; Hospital 12 de Octubre, Madrid, Spain; Hospital Universitario de Henares, Madrid, Spain

## Objetives

To evaluate the association of contrast type and dose in contrast-enhanced radiographic examinations with the incidence of CA-AKI in Spanish intensive care units (ICUs).

## Materials and Methods

Prospective multicenter-study in 34 Spanish ICUs covering a 4-month period (from December 15th, 2012 to March 15th, 2013). Research endorsed by the Spanish Society of Intensive, Critical and Emergency Care Medicine (SEMICYUC). From 1035 initial cases, exclusion criteria of uncompleted demography data and renal depuration yielded a final total of 1012 patients.

CA-AKI is defined as an absolute increase of 0.5mg/dl or 50% relative increase of serum creatinine 48-72 hours after contrast administration. Statistical analysis through multiple logistic regression with CA-AKI as dependent variable performed with software R3.1.2 for OsX. Included variables comprise those with p < 0.1 in univariant tests and those related to CA-AKI in literature. Results are expressed as average (standard error).

## Results

The most frequent radiographic exam was coronary angiography in 46.6% of the patients. Computed tomography (CT)/angio-CT were performed in 45.6% of them, and 7% underwent other explorations. The most frequent types of administered contrast were iso-osmolar in 52.2% of cases and hypo-osmolar in 43.8%. Iso-osmolar contrast was used in 50% of the coronary angiographies, in 51.8% of the CTs and and in 72.5% of the other explorations (p < 0.001).

The average contrast volume was different in each type of study: 178.2 (4.4) ml in coronary angiography, 164(10) ml in other explorations and 120.4 (1.8) ml in CT/angio-CT (p < 0.001), but it was not different according to the type of contrast: 148.2 (3.4) ml with iso-osmolar, 151(3.8) ml with hypo-osmolar and 149.4(15.7) ml with hyper-osmolar (p > 0.005). Incidence of CA-AKI was lower in coronary angiography (7.9%) than in CT (16.1%) and in other explorations (15.9%), p < 0.001, but neither the volume nor the type of contrast were associated with the development of CA-AKI.

Mortality was also lower in coronary angiography (5.3%) than in CT (21.5%) and other explorations (13%), p < 0.001, but it was independent of the volume and type of the contrast media (13.3% for iso-osmolar, 13.5% for hypo-osmolar and 14.6% for hyper-osmolar, p > 0.005.

## Conclusions

In our study, the incidence of CA-AKI and the mortality were independent of the type and volume of contrast. These findings are not explained by the different profiles (comorbidities) of the patients to whom the contrasts are administered.

